# Single-cell analysis of peptide expression and electrophysiology of right parietal neurons involved in male copulation behavior of a simultaneous hermaphrodite

**DOI:** 10.1007/s10158-015-0184-x

**Published:** 2015-12-06

**Authors:** Z. El Filali, P. A. C. M. de Boer, A. W. Pieneman, R. P. J. de Lange, R. F. Jansen, A. Ter Maat, R. C. van der Schors, K. W. Li, N. M. van Straalen, J. M. Koene

**Affiliations:** Department of Ecological Science, Faculty of Earth and Life Sciences, Vrije Universiteit, Amsterdam, The Netherlands; Department of Neurosciences, Faculty of Earth and Life Sciences, Vrije Universiteit, Amsterdam, The Netherlands; Max-Planck-Institute for Ornithology, Behavioral Neurobiology, Seewiesen, Germany

**Keywords:** FMRFamide, Hermaphroditism, *Lymnaea stagnalis*, MALDI-TOF-MS, Male mating, Neuropeptides, Right parietal ganglion, Penial nerve

## Abstract

Male copulation is a complex behavior that requires coordinated communication between the nervous system and the peripheral reproductive organs involved in mating. In hermaphroditic animals, such as the freshwater snail *Lymnaea stagnalis*, this complexity increases since the animal can behave both as male and female. The performance of the sexual role as a male is coordinated via a neuronal communication regulated by many peptidergic neurons, clustered in the cerebral and pedal ganglia and dispersed in the pleural and parietal ganglia. By combining single-cell matrix-assisted laser mass spectrometry with retrograde staining and electrophysiology, we analyzed neuropeptide expression of single neurons of the right parietal ganglion and their axonal projections into the penial nerve. Based on the neuropeptide profile of these neurons, we were able to reconstruct a chemical map of the right parietal ganglion revealing a striking correlation with the earlier electrophysiological and neuroanatomical studies. Neurons can be divided into two main groups: (i) neurons that express heptapeptides and (ii) neurons that do not. The neuronal projection of the different neurons into the penial nerve reveals a pattern where (spontaneous) activity is related to branching pattern. This heterogeneity in both neurochemical anatomy and branching pattern of the parietal neurons reflects the complexity of the peptidergic neurotransmission involved in the regulation of male mating behavior in this simultaneous hermaphrodite.

## Introduction

To ensure reproductive success, animals developed various strategies that guide them in choosing a partner, with the best genetic material, to mate with. This screening process, known as sexual selection, can take place pre- or post-copulatory in any mating species (Parker 1970; Koene 2012). Mating behavior, in general, shows a complex sequence of events accurately coordinated through specific neuronal networks that allow a precise chemical communication between neurons and their targets. To achieve this complex interaction, neurons use different signaling molecules, e.g., neuropeptides which play a crucial role in the regulation and modulation of many characteristics of reproductive behaviors in animals (Dornan and Malsbury 1989; El Filali et al. 2006; Koene 2010). A necessary step toward the comprehension of the different sequences of this behavior is the description of the neuronal connectivity between the different brain regions involved and the identification of their chemical messengers. In simultaneously hermaphroditic animals, the neural communication elevates its complexity, since the brain controls male and female behaviors within a single individual, while switching from one sexual role to the other.

Male mating in the pond snail, *Lymnaea stagnalis*, was the focus of many multidisciplinary studies aimed at getting a deep insight into the regulation of this behavior. It has been studied in great detail and at different levels: behavioral (van Duivenboden 1984; de Boer et al. 1996; Koene and Ter Maat 2005, 2007), electrophysiological (de Boer et al. 1997), biochemical (Li et al. 1992; 1995), immunohistochemical (de Lange et al. 1997, 1998a, b) and functional (van Golen et al. 1995a, b). Male mating is a complex behavior consisting of a series of stereotypic stages, movements and decisions that reflect an accurate communication between two partners that both have the ability to be male or female (van Duivenboden 1984; Koene and Ter Maat 2005). This complex communication is under the control of a relatively simple brain, characterized by a finite number of large, individually identifiable, neurons, making this animal a perfect model for single-cell analysis studies (Li et al. 1997; Jiménez et al. 1998; Koene 2010). The peripheral male reproductive organs are innervated by a single nerve called nervus penis (NP, penial nerve) that originates from the right cerebral ganglion and travels along the muscular preputium (the penis-carrying organ in snails). There, it divides into three branches innervating different parts of the reproductive tract known as the penial complex composed of the preputium carrying the penis, the vas deferens and the retractor muscles (Koene 2010; de Boer et al. 2010). The central neuronal network that innervates the penial complex includes various peptidergic neurons that can be divided in two categories: (i) clustered cells, forming the whole anterior and ventral lobe of the right cerebral ganglion and the I-cluster of the right pedal ganglion (de Boer et al. 1997; van Duivenboden 1984; Smit et al. 1992), and (ii) dispersed cells in the right pleural and parietal ganglia (van Duivenboden 1984). The clustered cells have been intensively studied, particularly the anterior lobe of the right cerebral ganglion (de Lange et al. 1997; Koene et al. 2000). However, the dispersed neurons, not being grouped and therefore not so easily visually defined, are less amenable for single-cell analysis, and their peptide contents remain largely unknown (Koene 2010; de Lange et al. 1998a). It is known that even neighboring neurons in the same ganglion may have different neuropeptide contents and hence different biological functions. Thus, it is important to apply a single-cell approach to reveal the chemical identity of dispersed cells that form part of the network controlling male mating. Therefore, our aim here is to provide a comprehensive description of these neurons in terms of peptide content and to relate that to their electrophysiological characteristics previously studied (de Lange et al. 1998a), in order to understand their involvement in the neural network underlying this behavior.

In terms of the neuropeptides involved, FMRFamide and related peptides are known to be important for male mating behavior (van Golen et al. 1995a) as well as for the regulation of many other physiological processes such as the heartbeat (Buckett et al. 1990) and egg laying (Brussaard et al. 1988, 1989). The primary transcript of the multiexon FMRFamide gene has been shown to be alternatively spliced, generating two different mRNAs (1 and 2) expressed in a differential and exclusive manner throughout the CNS of *Lymnaea* (Bright et al. 1993; Worster et al. 1998). The mRNA1 leads to the production of tetrapeptides, such as FLRF/FMRFamide and related peptides, while mRNA2 produces several heptapeptides, among which are GDPFLRFamide, SDPFLRFamide and related peptides, including the 35 amino acid peptide (Santama et al. 1995) which is also known as acidic peptide or abbreviated to DEILSR. Throughout this manuscript, we will refer to peptide products encoded by mRNA2 collectively as the heptapeptides.

Earlier studies on the right parietal ganglion using electrophysiological and immunocytochemical techniques revealed two groups of neurons: (i) spontaneously active neurons, containing heptapeptides, and (ii) silent neurons containing *Lymnaea* inhibitory neuropeptide (LIP) and no heptapeptides (de Lange et al. 1998a). However, the global peptidergic identity of these neurons remains largely unknown. A previous study, using matrix-assisted laser desorption/ionization time-of-flight mass spectrometry technique (MALDI-TOF-MS), demonstrated the feasibility of this technique in detecting peptides at single-cell level (El Filali et al. 2003). In the present study, we extended MALDI-TOF-MS analysis to include all the cells of the parietal ganglion that belong to the network regulating male copulation behavior, and combined this with retrograde staining and electrophysiology. In doing so, we aimed to create a map of the parietal neurons as well as a description of their neuropeptide content and neuroanatomy.

## Materials and methods

### Animals

Adult, laboratory-bred specimens of *L. stagnalis* (shell heights 28–33 mm) were used. The snails were bred under standard laboratory conditions (aerated, fresh, low-copper water at 20 ± 1 °C, 12-h/12-h light–dark cycle and fed on lettuce leaves).

### Retrograde staining

Cell bodies of the neurons that project into the penis nerve were identified by backfilling this nerve with nickel-lysine (the technique for axonal backfilling was modified after the procedure described earlier (Fredman 1987). The central nervous system (CNS) of *L. stagnalis* was pinned down in a dish containing saline solution (pH 7.9) composed of 53.3 mM NaCl, 1.7 mM KCl, 4.1 mM CaCl_2_, 1.5 mM MgCl_2_ and 5.0 mM Hepes buffer. The penis nerve was cut from the right cerebral ganglion and brought into a dish where the cut edge was immersed in a drop of nickel-lysine solution (1.7 g NiCl_2_–6H_2_O and 3.5 g of L-lysine free base in 20 ml H_2_O). To avoid diffusion, the nickel-lysine drop was enclosed in a thick mass of Vaseline. The preparation was completely immersed in saline and left at room temperature overnight. After at least 16 h of axon transport, the CNS was washed in fresh saline, and nickel was precipitated by adding 1 drop of saturated rubeanic acid (dithiooxamide) in ethanol per 1 ml of saline. After 15–20 min, the CNS was desheathed to expose the dark stained cells selected for MALDI-TOF-MS analysis.

### Sample preparation for MALDI-TOF-MS

After being first photographed to record their location, single stained neurons from the right parietal ganglion of the CNS were carefully removed with a glass pipette, ruptured in 0.3–0.5 µl of 0.1 % trifluoroacetic acid solution to allow the release of the neuropeptides and finally transferred individually onto a stainless steel MALDI sample plate. To each neuron, 0.3–0.5 µl of α-cyano-4-hydroxycinnamic acid matrix at 5 mg/ml in 50 % acetonitrile/50 % water containing 0.1 % trifluoroacetic acid was added. Afterward, the samples were air-dried and the whole plate was inserted into the mass spectrometer for peptide analysis using the Applied Biosystems 4700 Proteomics Analyzer with TOF/TOF optics (Medzihradszky et al. 2000; El Filali et al. 2003). This MALDI mass spectrometer uses a 200-Hz frequency tripled Nd:YAG laser operating at a wavelength of 355 nm.

### Electrophysiology

The extracellular electrical activity of the peripheral cut end of the penis nerve was recorded by using a glass microelectrode that was cut off at the appropriate diameter. Extracellular recordings of the three nerve branches of the penis nerve, NP1, NP2 and NP3, were made via *en passant* electrodes. The three branches project to different parts of the male copulation apparatus. NP1 projects toward the base of the preputium where one or more small branches split off. The main trunk of the NP1 projects beyond the preputium and runs along the vas deferens toward the prostate gland. NP2 projects to the preputium. A network of fine branches can be seen on the surface of the preputium. NP3 projects toward the penis sheath. Preparations for the extracellular recordings of the three nerve branches consisted of the CNS, preputium and intact nerve branches. The *en passant* electrodes were made of stainless steel fine wire of 25 µm in diameter. This recording technique has been described elsewhere (Hermann et al. 1994). In short, the fine wires are placed around the nerve and fixed in place by dental impression material (Reflect, Kerr). In some preparations, the NP2 had numerous projections toward the preputium which made it impossible to place an electrode. In these preparations, the recording electrode was placed more anterior to the location where NP2 and NP3 are still united. This resulted in recordings from NP1, NP2 + 3 and NP3.

Intracellular recordings of a total of 72 neurons in the right parietal ganglion of 5 preparations were made with glass microelectrodes (resistance 5–50 MΩ), which were filled with 0.5 M KCl. Only neurons which were expected to have an axon in the penis nerve, as deduced from their position seen in the retrograde staining of other preparations, were impaled. On average, 14 neurons were recorded per preparation. Action potentials were evoked by using a supra-threshold depolarizing current to verify whether the impaled neuron had an axon in the penis nerve or one of its branches (i.e., NP1, NP2 or NP3). Each impaled neuron was depolarized several times in order to assess whether the response in the nerve branch had a consistent latency.

## Results

### Direct mass profiling of single neurons of the right parietal ganglion

**Fig. 1:**
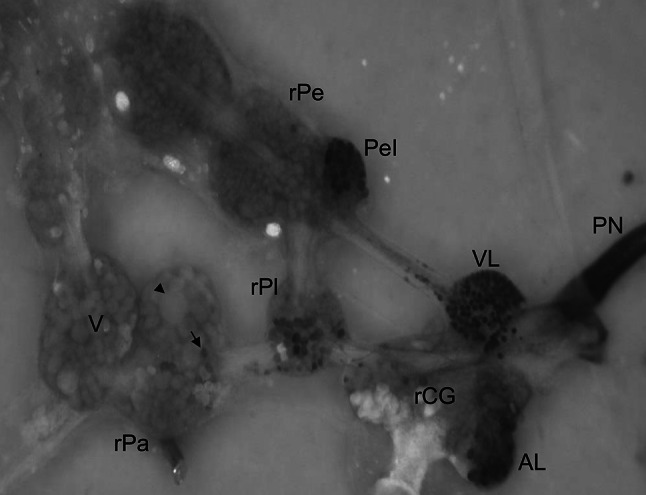
Dorsal view of a *Lymnaea stagnalis* central nervous system preparation after retrograde filling of the penial nerve with the nickel-lysine, showing the neuronal network regulating male mating. The cerebral commissure was cut after backfilling, and the cerebral ganglia placed to the sides to expose the pedal, pleural, and parietal ganglia. Retrograde-stained neurons appear bluish black. The *arrow* indicates an example of one of the dispersed cells in the right parietal ganglion that was selected for MALDI-TOF-MS analysis. The *arrowhead* indicates the giant neuron, right parietal dorsal one (RPD1). Different CNS backfills showed a variability of 5–10 stained neurons. Abbreviations: *AL* anterior lobe, *rPe* right pedal ganglion, *PeI* cluster I of pedal ganglion, *PN* penial nerve, *rPa* right parietal ganglion, *rPl* right pleural ganglion, *rCG* right cerebral ganglion, *V* visceral ganglion, *VL* ventral lobe

To establish the chemical identity of the dispersed neurons in the right parietal ganglion, neurons sending their axons to the penial nerve were first retrogradely stained to visualize them and make them accessible for MALDI-TOF-MS analysis. The retrograde staining reproducibly showed a neuronal network composed of (i) clusters of neurons in the anterior and ventral lobe of the right cerebral ganglion and the Ib cluster of the right pedal ganglion, and (ii) dispersed cells localized mainly in pleural and parietal ganglia (Fig. 1). To determine their peptidergic content, stained parietal neurons (15–30 cells) were removed and transferred to MALDI-TOF-MS. The analysis of the resulting spectra, showing ion species with masses between 400 and 3000 Da, revealed a complex pattern of peptide profile ranging from a total overlap to a mostly differential expression.

**Fig. 2:**
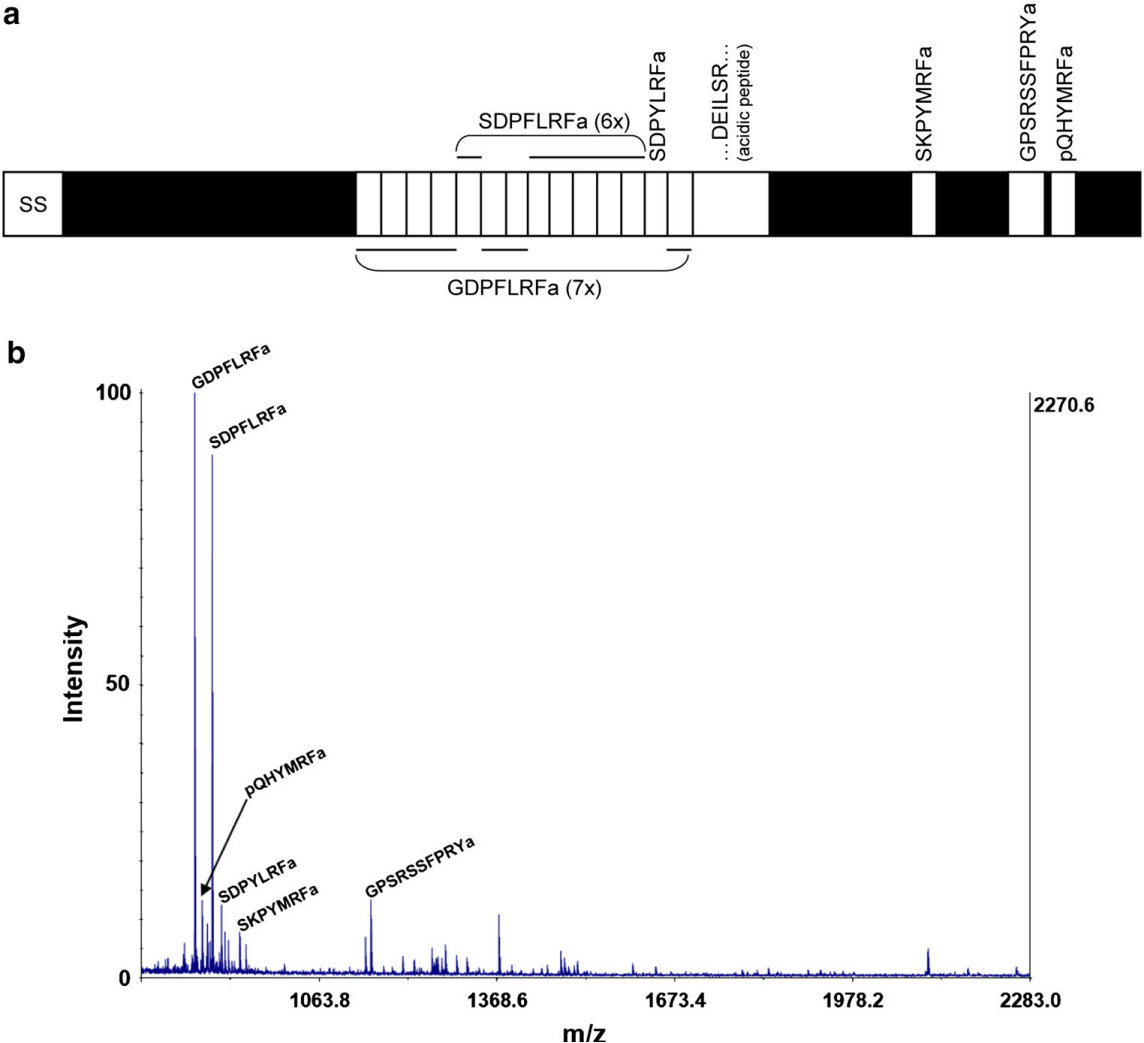
MALDI-TOF-MS analysis of the right parietal neurons containing heptapeptides (HP neurons). **a** Predicted protein precursor encoded by mRNA2 (the splicing product of the primary transcript of the FMRFamide gene) showing the putative neuropeptides contained within the precursor. Note that we assumed that the DEILSR containing 35 amino acid peptide (acidic peptide) is not cleaved into two smaller peptides. **b** Mass spectrum of a single retrograde-stained parietal heptapeptide-containing neuron. Note that all the ion species with molecular mass corresponding to those of predicted peptides from the FLRF precursor were detected: GDPFLRFamide, 850.40 Da; SDPFLRFamide, 880.41 Da; SDPYLRFamide, 896.40 Da; pQHYMRFamide, 863.34 Da; SKPYMRFamide, 927.42 Da and GPSRSSFPRYamide, 1152.51 Da. Unlabeled peaks correspond to unknown neuropeptides (*Novel*) that need to be further chemically characterized. All mass values quoted are for the [M + H] ion. Percent intensity refers to the relative signal intensity. The most intense peak (base peak) corresponds to GDPFLRFamide and is represented as 100 %. All other peaks are, therefore, represented as a proportion of the base peak intensity. The counts on the right-hand vertical axis show the absolute intensity

**Fig. 3:**
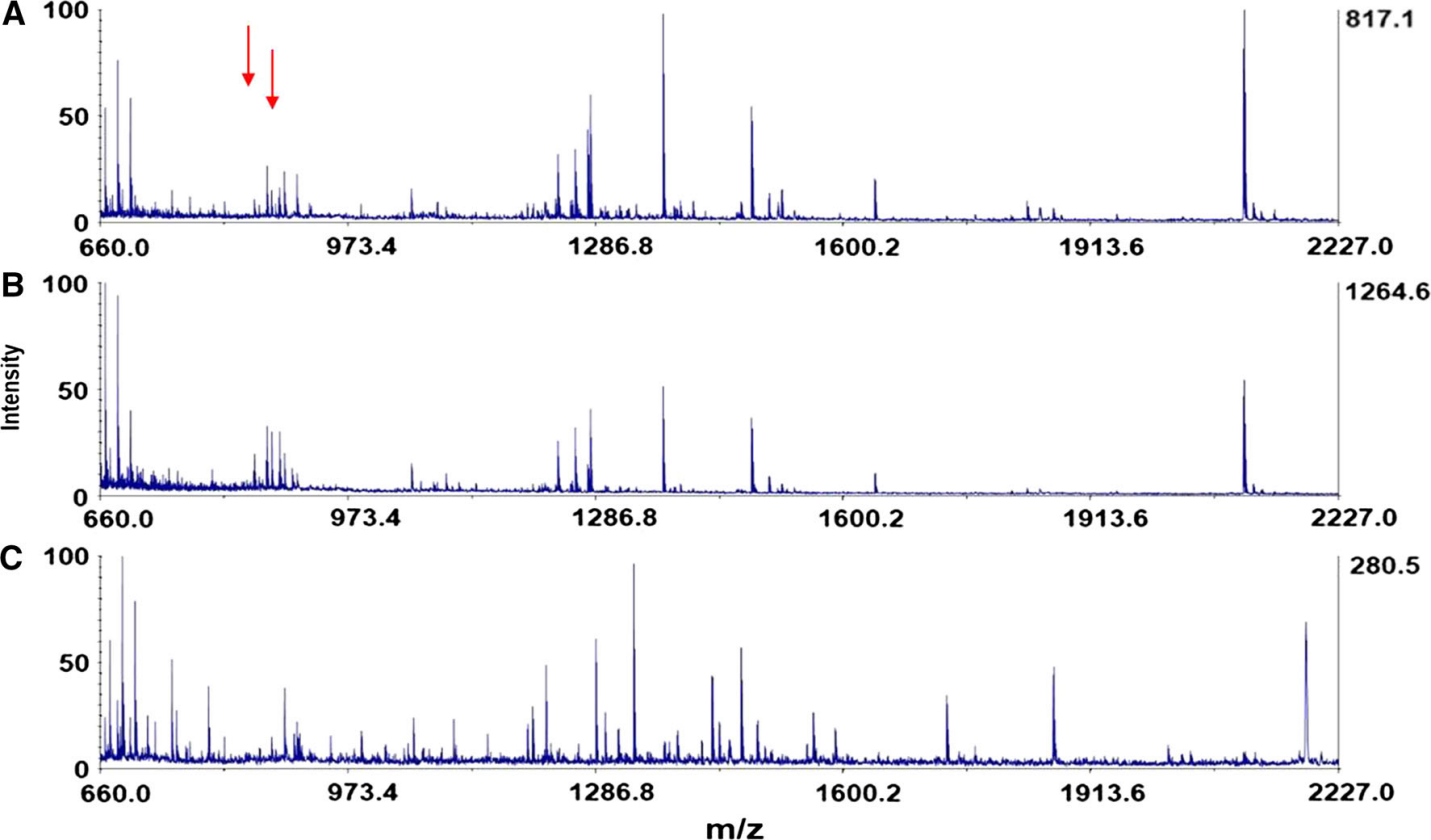
MALDI-TOF-MS analysis of three single retrograde-stained neurons containing no heptapeptides and located in different regions of the same right parietal ganglion (nonHP neurons). Note the total overlap in peptide content of the two spectra shown in A and B and the mostly differential expression of neuropeptides in spectrum C. The *arrows* indicate the predicted place for the two dominant heptapeptides (S/GDPFLRFamide) absent in these neurons (color figure online)

Based on the presence or absence of FMRFamide-related peptides in the right parietal ganglion, two types of neurons can be distinguished: neurons containing the heptapeptides and the related peptides (FLRFamide) encoded by transcript 2 of the FMRFamide gene (referred to as HP neurons; Fig. 2) and neurons lacking the transcript 2 peptides (referred to as nonHP neurons; Fig. 3). The mass spectrum of the HP neurons shows ions with masses corresponding to FLRFamide peptides encoded by the transcript 2 of the FMRFamide gene (Fig. 2a): GDPFLRFamide, 850.40 Da; SDPFLRFamide, 880.41 Da; SDPYLRFamide, 896.40 Da; pQHYMRFamide, 863.34 Da; SKPYMRFamide, 927.42 Da and GPSRSSFPRYamide, 1152.51 Da (Fig. 2b). Next to these predicted peptides, several unknown molecules corresponding to the unlabeled peaks are co-expressed with the heptapeptides. These putative neuropeptides need to be further identified in future research (see Fig. 2b; Table 1).

**Table 1:** List of molecular masses (*M* < 1400 Da) of putative neuropeptides identified from the two types of neurons in the right parietal ganglion

HP-neuron	NonHP neurons		
	A	B	C
650	645.33	–	671.98
665.97	*665.96*	*665.96*	687.96
681.94	*681.9*	*681.9*	697.91
850.40*	*697.91*	*697.91*	703.93
863.34*	*750.34*	–	*750.34*
880.41*	*870.34*	*870.34*	797.35
896.40*	*892.95*	*892.95*	893.34
908.4	*908.93*	*908.93*	908.9
927.42*	*1054.02*	*1054.02*	1056.41
938.41	*1239.54*	*1239.54*	1107.47
1143.4	*1261.51*	*1261.51*	*1207.59*
1152.51*	*1280.58*	*1280.58*	1224.51
*1207.59*	*1372.51*	*1372.51*	1287.49
1257.55	*1394.56*	*1394.56*	1299.48
*1280.59*			1335.54
1299.6			
1317.56			

The columns distinguish the main types, heptapeptide-containing neurons (HP-neuron) and nonheptapeptides-containing neurons (nonHP neurons). In bold are the masses of the FMRF-related peptides shown in the MALDI-MS spectra (Fig. 2). In italics are the masses that overlap between the different neurons. Note the overlap between nonHP-neuron A and B and the large difference in peptide contents between these two neurons and nonHP-neuron C (which correspond to the three panels in Fig. 3)

* El Filali et al. (2003)

The mass spectra of the nonHP neurons show that they neither express transcript 2 of the FMRFamide gene nor transcript 1 (Fig. 3; Table 1). The spectra of their peptide profile contain many ion species with masses that do not coincide with any known neuropeptides. These neurons show more diversity in their peptidergic identity, varying from a total overlap (e.g., the cells in Fig. 3a, b) to a mostly differential expression (e.g., Fig. 3c; Table 1). The ratios among the molecular ion species across the cells with a total peptidergic overlap remain constant, suggesting that the corresponding putative peptides are processed from the same precursor (Fig. 3a, b).

**Fig. 4:**
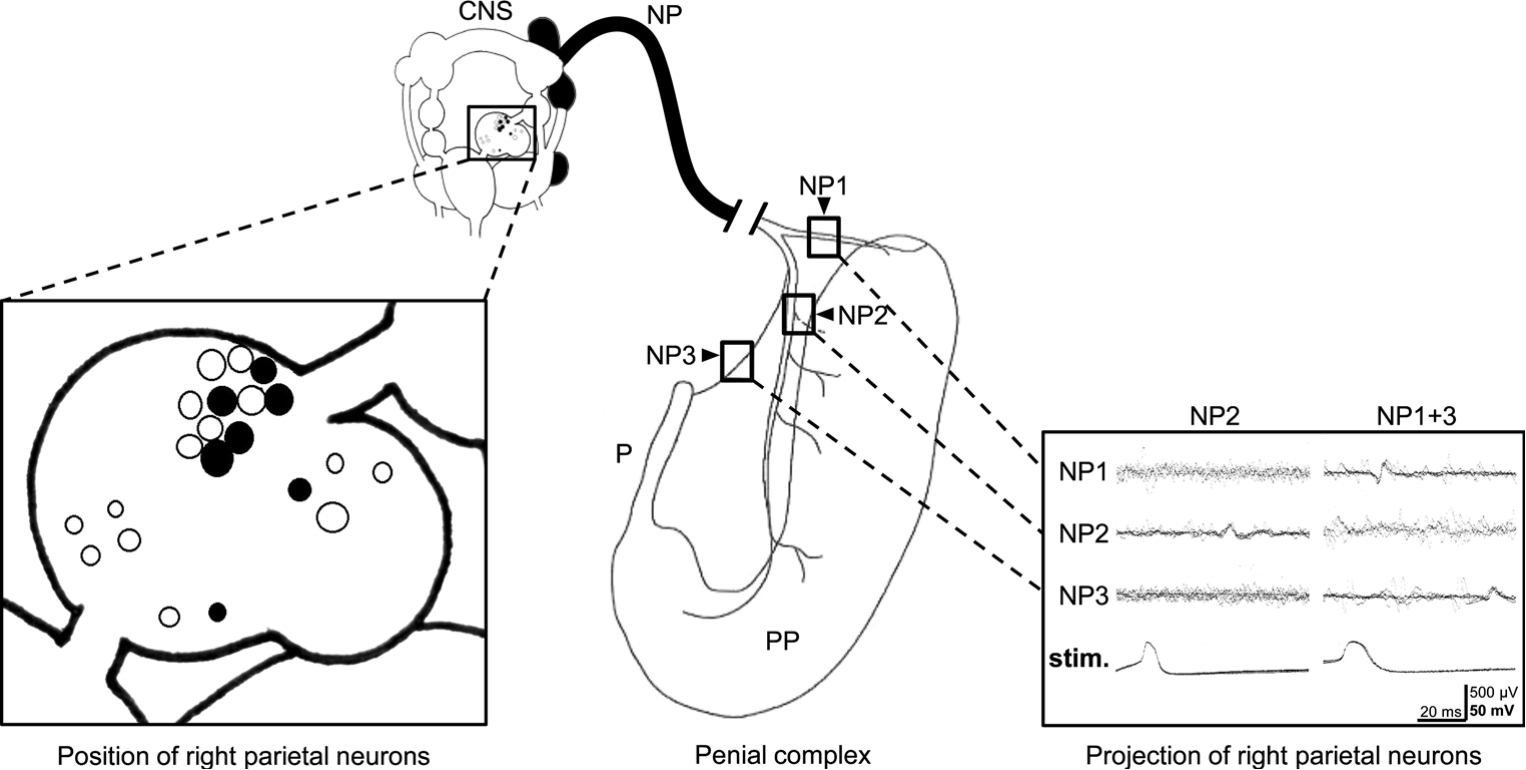
Neural projections and neuropeptide mapping of the right parietal neurons innervating the copulatory apparatus of *Lymnaea stagnalis*. The enlarged parietal ganglion (in the box) shows the heptapeptide-expressing neurons (HP neurons, *solid circles*) and the nonheptapeptide-expressing neurons (nonHP neurons, *open circles*). The *dark parts* in the central nervous system (CNS) correspond to the three other neuronal clusters that are relevant in this context (anterior and ventral lobes of the right cerebral ganglion and the Ib cluster of the right pedal ganglion, also shown in Fig. 1). The *right panel* shows six superimposed stimuli (stim.) of the same neuron and the response as it was measured in the three different nerve branches (NP1, NP2, NP3). As indicated above the traces, the left neuron only had an axon projecting into NP2, the right neuron had axons in both NP1 and 3. Abbreviations: *NP* penis nerve, NP1, NP2 and NP3, the three branches of the penis nerve; *P* penis, *PP* preputium

In Fig. 4, we summarize these data in a schematic map of the neurons present in the right parietal ganglion that are part of the network regulating the male behavior. We mapped the location of the neurons based on whether they contained heptapeptides or not.

### Neuronal projections of parietal neurons into the penial nerve

To characterize the neurons of the right parietal ganglion in terms of their neuroanatomy, intracellular recording of the neurons was combined with extracellular recordings of the penis nerve. The penis nerve divides into three branches: NP1, NP2 and NP3. Simultaneous recordings of these three branches were combined with intracellular recordings (*N* = 5 preparations) to investigate the projection of the neurons to the different parts of the male copulation apparatus. Figure 4 illustrates the branching pattern of the penial nerve, in which the mapped right parietal neurons project. Of the 72 neurons impaled, 23 had an axon in one or two branches of the penis nerve. Most of these cells were located adjacent to the right pleuro-parietal connective on the medio-lateral side of the right parietal ganglion. The projections of the 23 neurons were found primarily in NP2: Seven neurons projected selectively in the NP2, nine neurons projected in both NP2 and NP1, and two neurons projected in both NP2 and NP3. Of the remaining 5 neurons, two projected selectively in the NP1, one selectively in NP3 and two projected in both the NP1 and NP3 (Table 2). The neurons projecting solely into NP1 or NP3 or both into NP1 and NP3 were always silent (*N* *=* 5 neurons). The projection patterns of the neurons projecting into NP2, or NP2 and another branch, were not strictly correlated with their electrical activity. These neurons were either electrically silent, some showing an occasional action potential, or were spontaneously active. All 23 neurons had an orange to pale orange color. White neurons were often located near the neurons with an axon in the penis nerve, but none of these impaled white neurons had an axon branch in the penis nerve.

**Table 2:** Detailed branching pattern of neurons in the parietal ganglion into the nervus penis (NP)

	NP1	NP1 + 2	NP2	NP2 + 3	NP3	NP3 + 1
Observed (*N* = 23)	2	9	7	2	1	2
Spontaneous activity	No	Yes/No	Yes/No	Yes/No	No	No

These cells are involved in the regulation of male behavior of *Lymnaea stagnalis*. The observed numbers indicate the number of cells observed with a projection in one or more of the branches of the penial nerve (NP1, NP2, and/or NP3). The lower row also indicates whether spontaneous activity was recorded in these cells

## Discussion

Using a multidisciplinary approach combining the analytical power of MALDI-TOF-MS with neuronal labeling, we partially analyzed the peptide content of the right parietal neurons regulating male mating and identified some of the prominent peaks as the heptapeptides. As shown in Fig. 4, most of the identified neurons are located in the anterior dorsal side of the ganglion, a region known to contain a population of 15–18 neurons called group B, characterized by their shared morphological and electrical properties (Benjamin and Winlow 1981). However, although they were previously described in detail and carefully mapped, illustrations of the exact position of this group of neurons seem to vary (Bright et al. 1993; Benjamin and Winlow 1981; Kemenes et al. 1989). As a result of this, we can only conclude that at least some of the neurons that we analyzed belong to the B group (see also El Filali et al. 2003).

MALDI-TOF-MS allowed us to learn more about the neuropeptide expression pattern of these neurons, showing a large range of variation in neuropeptide composition varying from a total overlap to a mostly differential pattern. Based on the peptide profile, two types of neurons could be distinguished: HP neurons which express the heptapeptides and nonHP neurons which do not. The peptide content of the nonHP neurons revealed the absence of the neuropeptides encoded by the transcript 2 of the FMRFamide gene identified earlier (El Filali et al. 2003; Santama et al. 1995). Previous studies have shown that the expression of the FMRF-related peptides is differential in the whole parietal ganglion (Worster et al. 1998). The foregoing corroborates earlier immunocytochemical findings using antibodies against DEILSR, a partial sequence of the 35 amino acid peptide encoded by mRNA2, which is used as a marker for the presence of heptapeptides encoded on the same transcript (Santama et al. 1995). We confirm the presence of this peptide in the HP neurons only. This earlier immunocytochemical study also showed that anti-DEILSR and anti-LIP (*Lymnaea* inhibitory peptide) are mutually exclusive in the backfilled neurons of the right parietal ganglion, suggesting that at least some of the silent, DEILSR-negative parietal cells with projections into only the penis nerve are LIP-containing cells (de Lange et al. 1998a). However, our MALDI-TOF-MS data do not support this hypothesis since they did not confirm the presence of LIP in the nonHP neurons. Rather, our data are in agreement with work revealing the localization of the LIP-containing neurons in the ventral lobe of the right cerebral ganglion (Smit et al. 2003).

The fact that DEILSR-immunopositive axons have been found in several male reproductive organs (the preputium, penis sheath, the vas deferens and the prostate gland) (de Lange et al. 1998b) indicates that the HP neurons are involved in the fine coordination of male copulation (e.g., eversion of the preputium and penis and semen transport). This is also in agreement with the heptapeptides S/GDPFLRFamide having been shown to have a relaxing effect on the penis retractor muscle (PRM), involved in male copulation (van Golen et al. 1995a). In view of this inhibitory effect of the heptapeptides on its targets, it seems contradictory to assume that these neuropeptides are responsible for the contraction of the PRM needed for keeping the preputium inside the body wall of the snail to prevent male copulation activity (de Boer et al. 2010). However, since the heptapeptides are co-expressed with other putative neuropeptides (Fig. 2b), one could speculate that the excitatory effect of the HP neurons that lead to the contraction of the PRM is due to some other co-expressed neuropeptides that might counteract the inhibitory effect of the heptapeptides on the PRM. This hypothesis is in agreement with the results of earlier pharmacological studies which revealed that a single interneuron could have excitatory and inhibitory effects on different target cells (Skingsley et al. 1993).

The two types of neurons distinguished in this study differ not only in their peptide contents but also in their electrical activity and branching pattern, as partly published earlier (de Lange et al. 1998a). The nonHP neurons are found to be electrically silent and to have projections only into the penis nerve. The HP neurons, in contrast, were generally found to be electrically spontaneously active and to have multiple axonal projections to several nerves including the penis nerve. When combining this previous work with the electrical recordings presented here, an interesting pattern emerges. We find that neurons sending their axons to NP1, NP3 or both are exclusively silent. Neurons that send their axons to NP2 (either combined with an axon in NP1 or NP3) can either be silent or spontaneously active. The study by de Lange et al. (1998a, b) showed that nonDEILSR neurons (i.e., what we now call nonHP neurons) with a projection in the NP2 as well as another nerve were spontaneously active. All of this is summarized in Table 3, which combines our findings with the previous work.

**Table 3:** Summary of the characteristics of the right parietal neurons (RPa) involved in male mating

RPa cell type	Electrical activity	DEILSR^a^	Heptapeptides	NP branching^a^
HP neurons	Spontaneously active	Yes	Yes	NP2+ other nerves
NonHP neurons	Silent	No	No	only NP

The columns indicate whether the electrical activity of the two types of neurons was spontaneous or silent, whether heptapeptides (and DEILSR) were detected, and whether the branching patterns of the cells include more than only the nervus penis (NP)

^a^The presence of DEILSR is based on immunohistochemical work reported in a previous study as is the branching pattern into different nerves than the nervus penis (de Lange et al. 1998a)

The functional significance of these projections is not yet clear, but it suggests that the heptapeptide-containing neurons of the right parietal ganglion are multifunctional. Interestingly, the spontaneously active neurons are always found to have a projection into NP2, which mainly innervates the preputium itself and might thus give a clue as to why they are active. Furthermore, some neurons were also found to have projections into other nerves: for example, the nervus analis, nervus cutaneous pallialis and the nervus pallialis internus (de Lange et al. 1998a). The latter is interesting because this innervates the osphradium, a structure responsible for the detection and regulation of the water osmolarity (Kamardin 1995). Such information might be relevant to the hydrostatic pressure within the animal, which is in turn relevant to the eversion of the preputium. Also, the nervus analis and nervus cutaneous pallialis have been previously shown to be involved in the innervation of the columellar muscle controlling the shell movement (Plesch et al. 1974), which is also innervated by the ring neuron, the only identified interneuron in the reproductive network (Jansen and Ter Maat 1985).

In conclusion, we provide a partial peptidergic characterization of the dispersed cells in the right parietal ganglion, revealing a remarkable heterogeneity in both neurotransmission and neuronal projection and a close association between electrophysiology and peptide content. Clearly, next to these predicted molecules that have received attention in the past, many unknown peptides are expressed that would now need to be identified. Irrespective of their identity, the multitude of neuropeptides found in these neurons highlights the complexity of the peptidergic neurotransmission at single-cell level in the circuitry that regulates male mating in *Lymnaea stagnalis*. This complexity of regulation of the male reproduction likely extends to other simultaneous hermaphrodites, and it will now be especially interesting to investigate how this system is integrated with the regulation of female reproduction.
